# Clinical features and prognostic factors in patients with bone metastases from hepatocellular carcinoma after liver transplantation

**DOI:** 10.1186/1471-2407-11-492

**Published:** 2011-11-22

**Authors:** Jian He, Zhao-Chong Zeng, Jia Fan, Jian Zhou, Jing Sun, Bing Chen, Ping Yang, Bin-Liang Wang, Bo-Heng Zhang, Jian-Ying Zhang

**Affiliations:** 1 Department of Radiation Oncology, Zhongshan Hospital, Fudan University, Shanghai 200032, China; 2 Liver Cancer Institute, Zhongshan Hospital, Fudan University, Shanghai 200032, China; 3 Department of Medical Statistics, Zhongshan Hospital, Fudan University, Shanghai 200032, China

**Keywords:** Transplantation, Hepatocellular carcinoma, Bone metastases, Radiotherapy

## Abstract

**Background:**

Little is known about the clinical features and prognostic factors of bone metastases of hepatocellular carcinoma (HCC) following liver transplantation (LT).

**Methods:**

All adult patients undergoing LT from 2001 to 2010 were reviewed. Patients with HCC bone metastases after LT received external beam radiotherapy(EBRT) during this period. Demographic variables, laboratory values, and tumor characteristics were determined before LT and EBRT. Total radiation dose ranged from 8 to 60 Gy(median dose 40.0 Gy).

**Results:**

The trunk was the most common site of bone metastases with finding of expansile soft-tissue masses in 23.3% of patients. Overall pain relief from EBRT occurred in 96.7% (29/30). No consistent dose-response relationship was found for palliation of with doses between 30 and 56 Gy (*P *= 0.670). The median survivals from the time of bone metastases was 8.6 months. On univariate and multivariate analyses, better survival was significantly associated with a better Karnofsky performance status (KPS) and well-controlled intrahepatic tumor, but not with lower alpha-fetoprotein levels. The median time from LT to bone metastases was 7.1 months. Patients exceeding the Shanghai criteria presented with bone metastases earlier than those within the Fudan criteria. Patients with soft-tissue extension always had later bone metastases. The majority of deaths were caused by liver failure due to hepatic decompensation or tumor progression.

**Conclusion:**

The prognostic factors of bone metastases of HCC following LT are KPS and well-controlled intrahepatic. Even though survival is shorter for these patients, EBRT provides effective palliation of pain.

## Introduction

Over the last 30 years, liver transplantation (LT) was established as a durable therapy for all forms of end-stage liver disease [1,2]. Early experience with LT for management of hepatocellular carcinoma (HCC) resulted in poor post-transplantation survival and high recurrence rates that were attributed to suboptimal patient selection. Currently, preoperative imaging criteria based on the size and number of tumors are used to select candidates for LT. The Model for End Stage Liver Disease scoring system introduced in 2002 now offers priority for patients with HCC within the conventional Milan criteria [3]. These criteria were expanded by the University of California San Francisco [4] and Shanghai criteria [5], and those with tumors that exceed these criteria are at higher risk for recurrence and/or metastases. Bone is the second most common site of extrahepatic tumor metastases following the lung [6,7].

The presence of bone metastases was shown to be an independent predictor of poor outcome in patients with HCC recurrence following transplantation [6]. However, the clinical features and prognostic factors in patients with bone metastases from HCC after LT are rarely reported [8]. In this study, we retrospectively analyzed 30 patients with bone metastases from HCC after LT in order to identify prognostic factors and to explore a effective treatment.

## Patients and methods

Eight hundred fifty-three consecutive patients who underwent LT for the management of HCC between April 2001 and June 2010 at the Liver Cancer Institute, Zhongshan Hospital, Fudan University were reviewed. Patients were identified from the Institute's prospectively collected database. A total of 30 patients undergoing external bean radiotherapy (EBRT) for bone metastases were identified among these LT patients. Ethics approval for the use of human subjects was obtained from the research ethics committee of Zhongshan Hospital, and informed consent was obtained from each patient.

Diagnosis of HCC was established by a combination of imaging studies and measurement of alpha-fetoprotein (AFP) levels prior to LT with final confirmation by explant pathology, without routine use of pretransplant tumor biopsy. Disease extent was determined by preoperative computed tomography (CT) or magnetic resonance imaging (MRI) performed within 3 months before LT. The two modalities showed consistent results. Extrahepatic metastasis was excluded based on chest and abdominal CT or MRI, and bone scintigraphy performed within 1 month before LT. Patients underwent classic orthotopic LT. Immunosuppressive therapy after LT consisted of a triple drug regimen of cyclosporine or tacrolimus combined with corticosteroids and/or mycophenolate mofetil [5]. Patients with HCC were classified as having tumors either meeting the Milan criteria [9], beyond Milan criteria but within the University of California San Francisco criteria [10], or within the Shanghai criteria. For Shanghai criteria, we determined that expansion of Milan criteria to include: a solitary lesion ≤9 cm in diameter, no more than three lesions with the largest ≤5 cm, a total tumor diameter ≤9 cm without macrovascular invasion, lymph node invasion and extrahepatic metastasis [5].

Diagnosis of bone metastases was based on the history of HCC, presence of symptoms, and radiologic imaging studies. The confirmation of bone metastases by histologic testing was not recommended in this study. All patients were required to undergo technetium-99 m bone scintigraphy, which is the best method for screening patients at risk for bone metastasis and is useful for evaluating the extent of metastatic bone disease. Bone scintigraphy is not specific for metastatic disease, and positive findings must often be confirmed using other imaging studies [11]. A confirmatory study (MRI, as a first choice, or CT) was especially important in determining the presence of soft-tissue extension, bone destruction, or spinal cord compression, and the extent of osteolytic or osteoblastic metastases [12]. Pure osteolytic metastasis was defined as bone destruction without new bone formation, as determined by CT scan or MRI, and no increased isotope uptake ("hot" spot) detected at the corresponding area on bone scan. Osteoblastic metastasis was defined as new bone formation visible by CT scan or MRI in the involved bone, with increased isotope uptake detected at the corresponding area on bone scan. Mixed osteolytic and osteoblastic lesions were the most common in patients with bone metastases from HCC [12]. The imaging diagnosis of bone metastasis was based on the combination with bone scintigraphy and computed tomography in 14 patients or magnetic resonance imaging in 16 patients. Positron emission tomography scanning to evaluate areas of increased metabolic activity was not routinely used in this study.

Indications for EBRT for bone metastases included pain, risk for pathologic fracture, and neurological complications arising from spinal cord compression and nerve root pain. Patients with multiple bone metastases, whose lesions caused pain or possibly spinal cord compression, were first considered for EBRT. Bone metastases status was recorded at the initial radiotherapy session. Radiation was delivered through a single posterior field or parallel opposed fields, depending on the location and depth of lesions base on CT or MRI. Most therapy was provided with 6- or 15-MV photons; however, electron therapy was also selected for those with shallow lesions such as in the ribs, skull, or extremities. Radiation fields included gross tumor volume and 1- to 1.5-cm margins. In the case of vertebral bone metastases, radiation fields usually encompassed one normal vertebra above and below the metastatic lesions. If the lesions presented with concurrent soft-tissue extension, the radiation fields were enlarged on the basis of CT or MRI results. We scheduled the full radiation dosage at 46 Gy for the vertebral metastatic lesions and 50-60 Gy for soft-tissue involvement beyond the spinal cord, in daily doses of 2 Gy/fraction five times a week. Factors that necessitated a reduced dose were progressive primary disease, many lesions, poor performance status, adverse effects, and patient inconvenience during EBRT.

Well-controlled intrahepatic primary tumors were defined as follows: (1) after liver transplantation, follow-up enhanced CT or MRI (or both) did not show any new lesions during the periods of EBRT, and (2) after intrahepatic recurrence, the patients received TACE or radiofrequency ablation, lipiodol was deposited in the entire intrahepatic tumor, or destruction of tumor within the zone of ablation, the follow-up enhanced CT or MRI did not show any new lesions, including the edge of primary tumors, during the periods of EBRT. Otherwise, the patients were regarded as having uncontrolled primary lesions.

In this study, we assigned Dr. Jian He who is the first author in this paper to focus on this study for 10 years (from 2001 to 2010). He recorded the patients' clinical data and followed up every patient. The pain measurements were done pre- and post RT using a visual analogue scale, which is part of routine practice at our institution [12].

Pretreatment evaluation included a medical history and physical examination, complete blood cell count, serum chemistries, liver function tests, AFP for those who tested positive at the initial evaluation, chest X-ray, abdominal ultrasonography, bone scintigraphy, and enhanced CT or MRI (or both). Clinical monitoring was performed once a week. Liver function was estimated using the Child-Pugh classification, which was scored based on the levels of serum bilirubin, serum albumin, prothrombin time prolongation, presence or absence of ascites, and encephalopathy.

Two survival intervals were estimated in this study. One was survival after bone metastases, i.e., the interval from the date when bone metastases were confirmed to death or the last follow-up. The other was bone metastasis-free survival, which was defined as the time from the date of LT to the date bone metastases were confirmed. Cumulative survival rates were analyzed using Kaplan-Meier curves. The median follow-up from EBRT is 9.7 (range: 0.83-63.87) months.

The trend χ^2 ^test was used to compare dose-response results. The Pearson χ^2 ^test or Fisher's exact probability was calculated to measure correlation among the variables. Potential prognostic factors were evaluated with respect to survival. For multivariate analysis, all variables were entered using the Backward-Wald method. Logistic regression was used to predict a categorical variable from a set of predictor variables in bone metastasis-free survival after LT. A *P *≤ 0.05 was considered statistically significant. All calculations were performed using SPSS 13.0 for Windows (SPSS, Chicago, IL).

## Results

### Clinical features

The cohort included 26 men and 4 women (ratio 6.5:1), with a mean age of 49.6 ± 10.0 years (range, 30-69 years). EBRT was performed on a total of 49 metastatic sites. The trunk was the most common site of bone metastases, with metastases most frequently occurring in the thorax (43.4%), pelvis (24.5%), and lumbar spine (9.4%), as shown in Table 1. All 30 patients in this cohort had a combination of both osteolytic and osteoblastic components. No patient appeared to have purely osteolytic lesions, for which false-negative readings were presented in the bone scintigraphy. Bone metastases with expansile soft-tissue masses were found in seven patients (23.3%).

**Table 1:** Sites of bone metastases in 30 HCC patients after liver transplantation

Bone sites	Present sites	Radiation sites
Skull	3 (5.7%)	3 (6.1%)

Cervical vertebrae	3 (5.7%)	2 (4.1%)

Upper limb		

Humerus	1 (1.9%)	0 (0%)

Thorax		

Thoracic vertebrae	8 (15.1%)	8 (16.3%)

Rib	12 (22.6%)	11 (22.4%)

Sternum	3 (5.7%)	3 (6.1%)

Lumbar vertebrae	5 (9.4%)	5 (10.2%)

Pelvis		

Sacrum	2 (3.8%)	1 (2.0%)

Sacroiliac joint	2 (3.8%)	2 (4.1%)

Ilium	4 (7.4%)	4 (8.2%)

Ischium	1 (1.9%)	1 (2.0%)

Acetabulum	4 (7.4%)	4 (8.2%)

Lower limb		

Femur	5 (9.4%)	5 (10.2%)

Total	53	49

### Response

Of 30 patients with bone metastases from HCC after LT, complete pain relief (CR) with EBRT occurred in nine (30.0%) patients and partial pain relief (PR) in 20 (66.7%). There was no response to EBRT in one patient who received 8 Gy/4 Fx radiation. The association between radiation dose and response was not significant (*X*^2 ^= 0.800, P = 0.670) in patients with CR and PR. It appears there is no consistent dose-response relation for the palliation of bone metastases (Table 2).

**Table 2:** Dose and response

Total dose	CR	PR	Total	*P *value
30 to < 40 Gy	1 (25.0%)	3 (75.0%)	4	0.670
	
≥40 to 46 Gy	6 (28.6%)	15 (71.4%)	21	
	
≥50 to 60 Gy	2 (50.0%)	2 (50.0%)	4	
	
Total	9	20	29	

Pearson's chi-square is used to assess P value

### Predictors of bone metastases before and after LT

**Figure 1:**
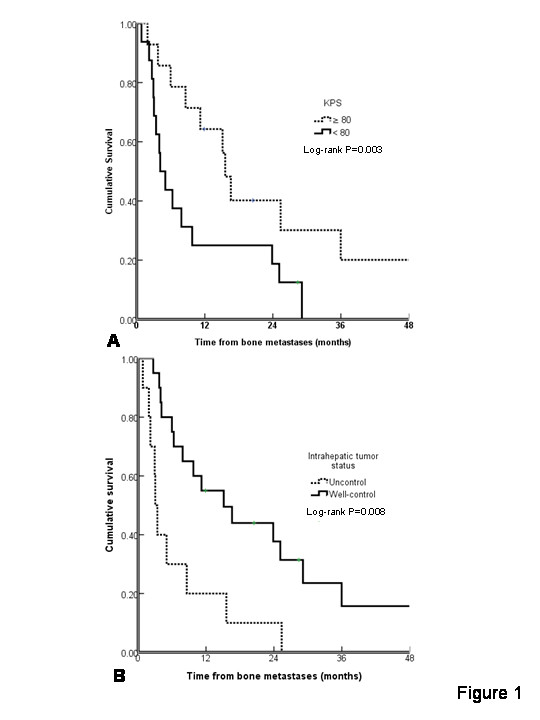
**Survival curves are shown based on a Karnofsky performance status and b intrahepatic tumor status**.

The 1-year, 2-year, 3-year, and median survivals from the time of LT were 70.0%, 38.6%, 31.6%, and 18.0 months, respectively. The 1-year, 2-year, and median survivals from the time of bone metastases were 39.7%, 24.4%, and 8.6 months, respectively. The impact of potential prognostic factors on survival is shown in Table 3. On univariate and multivariate analyses, better survival was significantly associated with a better Karnofsky performance status and well-controlled intrahepatic tumor as shown in Figure 1.

**Table 3:** Univariate and multivariate analysis of predictors of survival after bone metastases in 30 HCC patients undergoing LT

		**No**.	Survival status			P value	
			
			1-year	2-year	Median	Univariate	Multivariate
Pre-LT							

Gender	Female	4	50.0	0	2.7	0.241	0.428
	
	Male	26	42.3	29.0	8.6		

Age (years)	≤50	17	28.2	21.2	8.6	0.697	0.180
	
	>50	13	46.2	19.2	15.1		

HbsAg	-	3	0	0	5.0	0.190	0.201
	
	+	27	44.1	27.1	9.8		

Γ-GT	≤75	14	42.9	21.4	11.2	0.814	0.099
	
	>75	16	30.0	20.0	6.3		

AFP	≤400	16	37.5	23.4	8.6	0.738	0.557
	
	>400	14	34.3	17.1	6.3		

Child-Pugh Classification	A	22	26.5	21.2	6.3	0.240	0.099
	
	B	8	62.5	33.3	16.6		

Shanghai criteria	Exceeding	14	34.3	12.9	8.6	0.962	0.570
	
	Within	16	37.5	18.8	7.9		

UCSF	Exceeding	18	38.1	8.5	8.6	0.597	0.972
	
	Within	12	33.3	33.3	6.3		

Milan	Exceeding	20	50	20	11.2	0.939	0.364
	
	Within	10	20	20	4.1		

Interval between diagnosis of HCC and LT	≤0.5 year	17	35.3	22.1	8.6	0.947	0.976
	
	>0.5 year	13	36.9	18.5	9.8		

Post-LT							

KPS	≥80	14	56.3	30.1	15.6	0.032	0.003
	
	<80	16	18.8	12.5	4.1		

Γ-GT	≤75	11	53.0	31.8	23.9	0.116	0.385
	
	>75	19	26.3	14.0	6.3		

AFP	≤400	15	40.0	25.0	11.2	0.531	0.926
	
	>400	15	32.0	16.0	5.0		

Child-Pugh Classification	A	23	43.0	27.3	11.2	0.209	0.294
	
	B	7	14.3	0	6.3		

Soft-tissue extension	Absent	23	29.8	19.9	7.9	0.470	0.974
	
	Present	7	57.1	19.0	23.9		

No. of bone metastases	Solitary	14	50.0	25.7	15.6	0.245	0.456
	
	≥2 sites	16	23.4	15.6	7.9		

Intrahepatic tumor control	uncontrolled	10	20.0	10.0	3.0	0.003	0.008
	
	Well-controlled	20	55.0	31.4	15.1		

Concurrent metastases	Absent	15	38.9	15.6	4.1	0.565	0.170
	
	Present	15	40.0	24.0	9.8		

### Time from LT to bone metastases

**Figure 2:**
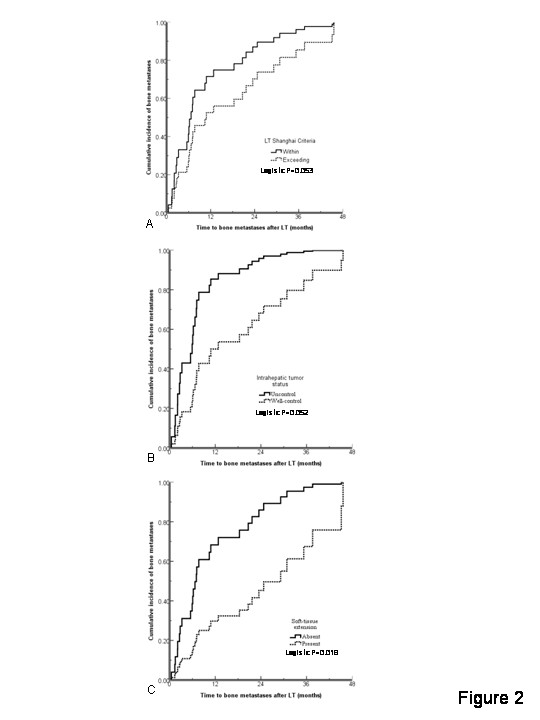
**The probability of bone metastases with the time to bone metastases after LT for different tumor status**.

The median time from LT to bone metastases was 7.1 months (mean time 14.5 months with range between 0.5 and 45.6 months). Patients who exceeded the Shanghai criteria presented with bone metastases earlier than those within Shanghai criteria (Logistic *P *= 0.053, Figure 2a). Patients with earlier bone metastases had higher incidence of intrahepatic uncontrolled tumor (Logistic *P *= 0.018, Figure 2b), but these were not related to concurrent distant metastases. Patients with soft-tissue extension always had later bone metastases as shown in Figure 2c and Table 4.

**Table 4:** Bone metastases free survival after LT

		Bone metasta ses-free survival		*P *value
		
		≤ 1 year	> 1 year	χ^2 ^test Logistic
Gender	Female	2 (6.7%)	2 (6.7%)	1.00
	
	Male	16 (53.3%)	10 (33.3%)	

Age	≤50	11 (36.7%)	6 (20.0%)	0.711
	
	>50	7 (23.3%)	6 (20.0%)	

HbsAg	-	3 (10.0%)	0 (0%)	0.255
	
	+	15 (50.0%)	12 (40.0%)	

Γ-GT	≤75	8 (26.7%)	6 (20.0%)	1.00
	
	>75	10 (33.3%)	6 (20.0%)	

AFP	≤400	9 (30.0%)	7 (23.3%)	0.722
	
	>400	9 (30.0%)	5 (16.7%)	

Child-Pugh Classification	A	14 (46.7%)	8 (26.7%)	0.678
	
	B	4 (13.3%)	4 (13.3%)	

Shanghai criteria	Exceeding	11 (36.7%)	3 (10.0%)	0.072 0.053
	
	Within	7 (23.3%)	9 (30.0%)	

UCSF	Exceeding	13 (43.3%)	5 (16.7%)	0.136
	
	Within	5 (16.7%)	7 (23.3%)	

Milan	Exceeding	13 (43.3%)	7 (23.3%)	0.694
	
	Within	5 (16.7%)	5 (16.7%)	

Interval between diagnosis of HCC and LT	≤0.5 year	8 (26.7%)	9 (30.0%)	0.141
	
	>0.5 year	10 (33.3%)	3 (10.0%)	

Soft-tissue extension	Absent	16 (53.3%)	5 (16.7%)	0.013 0.052
	
	Present	2 (6.7%)	7 (23.3%)	

No. of bone metastases	Solitary	6 (20.0%)	8 (26.7%)	0.135
	
	≥2 sites	12 (40.0%)	4 (13.3%)	

Intrahepatic tumor control	uncontrolled	9 (30.0%)	1 (3.3%)	0.045 0.018
	
	Well-controlled	9 (30.0%)	11 (36.7%)	

Concurrent distant metastases	Absent	10 (33.3%)	5 (16.7%)	0.710
	
	Present	8 (26.7%)	7 (23.3%)	

### Failure patterns and toxicity

At the end of this study, five patients (16.7%) were alive and 25 (83.3%) had died. The causes of death were liver failure due to hepatic decompensation, tumor progression, or both in 21 patients (84.0%); brain metastases in 2 patients (8.0%); lung infection in 1 patient (4.0%); and stroke in 1 patient (4.0%).

Acute radiation toxicity was mild or absent in all patients. If the radiation field involved the gastrointestinal area (such as bone metastases located between thoracic vertebra 10 and the sacrum), patients had mild loss of appetite and occasional nausea. Localized hair loss was found in patients with irradiation to the skull. Localized pigmentation change was found in the radiation fields. None of these adverse effects affected the timing or delivery of EBRT. No medical management was required for any radiation-associated toxicity.

## Discussion

A few articles have reported the natural history, effects of medical or surgical treatment, and predictors of survival in patients who develop recurrence of HCC after LT. There are no systematic reports on bone metastases from HCC after LT. Dr. Roayaie et al. previously reported a median survival of 5-6 months in 19 patients, which was the largest cohort of patients studied with bone metastases after LT [6]. Irrespective of other sites of metastasis, the presence of bone metastases was shown to be an independent predictor of poor outcome in patients with post-transplant HCC recurrence [6]. Treatments for bone metastases are aimed at palliating symptoms, and have the same modest role in the post-transplant setting as in non-transplant patients. Local radiotherapy provides effective palliation of painful skeletal lesions [12,13]. Survival and pain response rates are similar to those in patients without LT as we reported previously [12].

The clinical features of bone metastases from HCC after LT mimic other malignancies including those in HCC without LT, in which bone metastases most commonly affect the axial skeleton. The most common sites of bone metastases are the spine and ribs. However, bone metastases from HCC have their own characteristics. It is interesting to note that while nearly 40% of patients without LT had an accompanying hypervascular soft-tissue mass [12], only 23.3% of patients in this study showed accompanying hypervascular soft-tissue lesions. We do not know the factors contributing to the presence of expansile soft-tissue masses, but they are associated with the recurrence time between LT and bone metastases. Patients with soft-tissue extension always had delayed bone metastases (≥ 6 months).

Even though the treatment for bone metastases is the same in patients with and without LT, there are slight differences in both clinical features and prognosis. Poorer survival was found to be significantly associated with a higher AFP level in patients without LT [12]; however, the difference was not significant in patients with LT in this study. AFP is a serum marker that is elevated in 50-80% of patients with HCC. AFP is a significant independent predictor of survival as reported by many authors. Patients with normal AFP levels survive longer than those with elevated levels [14-16]. AFP can induce functional impairments in dendritic cells [17], resulting in these cells displaying an immature phenotype and/or defective function in peripheral blood of patients with HCC [18]. Priming of immune responses against AFP results in significant protective antitumoral T-cell responses in a mouse model [19]. All patients including AFP positive and negative patients are treated with immunosuppressive drugs to prevent rejection after LT. This may be useful for explaining the difference between the patients with and without LT.

There was a wide time range from LT to recurrence and in post-recurrence survival. In a series of 311 patients who underwent LT for HCC at Mount Sinai Medical Center, 57 patients had a median recurrence time of 12.3 months following transplantation [6]. Similar results were reported from Korea [20] and Spain [21]. In this study, the median time from LT to bone metastases was 7.1 months, which is shorter than in those with distant metastases beyond bone. Dr. Shin et al. reported that the median survival time after recurrence was 11.7 months, and the 1- and 3-year survival rates after recurrence were 52.8% and 15.8%, respectively [20]. After recurrence, the median survival was 7 months in 28 patients [7] and 8.7 months in 57 patients [6]. Our results showed that median survival after bone metastases is 8.6 months, which is between the times reported by the studies mentioned above. The prognosis of bone metastases after LT may be similar to those patients with metastases to other sites.

Limitations to our study are small patient numbers, a wide range of radiation doses (8-60 Gy), and lack of the incidence rate for bone metastases. Bone metastases from HCC itself rarely cause patient death, but bone metastases are a common cause of pain and other significant symptoms that are detrimental to quality of life. The differences in radiation dose did not affect survival. Patients with bone metastases were referred from surgeons to our department; and we did not know the exact number of patients with bone metastases who did not have symptoms. HCC recurrence after LT can be minimized or prevented by employing strict selection criteria. The incorporation of molecular data into clinical practice has the potential both to prevent recurrence through improved case selection [22], and to guide treatment by identifying patterns of gene expression that predict response to targeted therapies.

## List of abbreviations

HCC: Hepatocellular carcinoma; LT: Liver transplantation; EBRT: External beam radiotherapy; AFP: Alpha-fetoprotein

## Competing interests

The authors declare that they have no competing interests.

## Authors' contributions

ZZC organized the study, planned the experiments, performed the statistical analysis and helped to write the manuscript. HJ contributed to collect the clinical data and treat the most of patients with BM. FJ and ZJ are surgeons who participated in the design and coordination of the study in liver transplantation. SJ, CB, YP, and WBL are radiation oncologists who contributed to treat the patients with BM, and help Dr. He J to collect data. ZBH performed the statistical analysis, and drafted the manuscript. ZJY is a radiation physicist to design radiation fields and calculate radiation dose. All authors read and approved the final manuscript.

## Pre-publication history

The pre-publication history for this paper can be accessed here:

http://www.biomedcentral.com/1471-2407/11/492/prepub
